# Moving towards supraspinal TRPV1 receptors for chronic pain relief

**DOI:** 10.1186/1744-8069-6-66

**Published:** 2010-10-11

**Authors:** Enza Palazzo, Livio Luongo, Vito de Novellis, Liberato Berrino, Francesco Rossi, Sabatino Maione

**Affiliations:** 1 Department of Experimental Medicine, Pharmacology Division, The Second University of Naples, Naples, Italy; 2 Centro Interdipartimentale di Ricerca e Menagement, The Second University of Naples, Naples, Italy

## Abstract

Transient receptor potential vanilloid type 1 (TRPV1) receptor is a non selective ligand-gated cation channel activated by capsaicin, heat, protons and endogenous lipids termed endovanilloids. As well as peripheral primary afferent neurons and dorsal root ganglia, TRPV1 receptor is also expressed in spinal and supraspinal structures such as those belonging to the endogenous antinociceptive descending pathway which is a circuitry of the supraspinal central nervous system whose task is to counteract pain. It includes periaqueductal grey (PAG) and rostral ventromedial medulla (RVM) whose activation leads to analgesia. Such an effect is associated with a glutamate increase and the activation of OFF and inhibition of ON cell population in the rostral ventromedial medulla (RVM). Activation of the antinociceptive descending pathway via TPRV1 receptor stimulation in the PAG may be a novel strategy for producing analgesia in chronic pain. This review will summarize the more recent insights into the role of TRPV1 receptor within the antinociceptive descending pathway and its possible exploitation as a target for new pain-killer agents in chronic pain conditions, with particular emphasis on the most untreatable pain state: neuropathic pain.

## TRPV1 receptor: a member of TRP family channels

TRP ion channels described for first time in *Drosophila melanogaster *[1] are ion channels that respond to mechanical, thermal, chemical (i.e. acid, lipids) and many other stimuli coming from the extra and intracellular milieu [2-5]. The TRP channel family contains seven divisions: TRPC (canonical), TRPV (vanilloid), TRPM (melastatin), TRPA (ankyrin), TRPP (policystin) and TRPML (mucolipin) [2,6-8]. TRPV1, however, remains the most studied and best characterized TRP family member, due to the fact that it has been implicated in a wide variety of cellular and physiological processes, including noxious physical and chemical stimuli detection, making it a promising target for pain-relieving drugs acting exactly where pain originates.

The TRPV1 channel consists of six transmembrane domains assembled as homo or hetero-tetramers with each sub-unit contributing to the cation channel structure [9-11]. It is activated by capsaicin, the pungent ingredient found in the hot chilli pepper [12], resiniferatoxin (RTX), a highly irritant diterpene ester isolated from *Euphorbia resinifera *[13], noxious heat (> 43°C), low pH (5.2) [12,14], voltage [15,16] and various endogenous lipids such as anandamide which also activates cannabinoid type 1 (CB1) receptors, 12-hydroperoxy-eicosatetraenoic acid (12-HPETE) and N-arachidonoyl dopamine (NADA) [17-19]. Other natural compounds activating TRPV1 receptor are piperine found in black pepper, eugenol in cloves and zingerone in horseradish, allicin present in garlic and onion, gingerols present in raw ginger and shogaols, which are dehydration products of gingerols present in steamed ginger [20-26]. All these compounds are lipophilic and therefore bind to the intracellular surface of TRPV1 receptor [26]. Camphor is a natural compound that activates heterologously-expressed TRPV1 channels and potentiates TRPV1 currents in dorsal root ganglia (DRG) neurons at higher doses and at a different site from capsaicin. Camphor is used as a topical analgesic since it completely desensitizes the TRPV1 channel, through a vanilloid-independent mechanism and more rapidly than capsaicin [27]. TRPV1 is directly gated by noxious heat (> 43°C), which produces a sensation of pain through direct activation or through the efferent release of pro-inflammatory neuropeptides (neurogenic inflammation) [28]. Its expression on free nerve terminals in the skin allows us to detect nociceptive temperatures and facilitates its exposition to several modulators produced in response to inflammatory conditions or tissue damage that potentiate the channel's response to temperature. Thus under certain cellular conditions, such as inflammation and ischemia, TRPV1 receptor activation leads to pain under physiological temperature. The sensitivity of TRPV1 receptor also depends on membrane potential since the channel can open in the absence of capsaicin at room temperature (23°C) at depolarized potentials [29]. In addition, TRPV1 receptor is activated and sensitized by acidic pH; a condition that leads to pain during inflammation and ischemia [30,31].

## Peripheral and spinal TRPV1 receptor distribution

TRPV1 receptor has been found in both the peripheral and central nervous system within centres known for their role in pain detection, transmission and regulation, consistent with its key role in pain. Indeed, TRPV1 receptor is expressed in all sensory ganglia (DRG, TG, Vagal) and in small sensory C and Aδ fibers, which may contain various neuropeptides including substance P (SP) and/or calcitonin gene-related peptide (CGRP) [12,32-41]. These fibers terminate predominantly in lamina I and II of the superficial dorsal horn [42,43]. TRPV1 receptor is also expressed postsynaptically on lamina II cell bodies [43] and within the lateral collateral path, where the majority of visceral afferents terminate [44]. Spinal cord TRPV1 receptor labelling has mainly been found in the lumbar segment L4-L6 and preferentially expressed by visceral afferents [44]. TRPV1 receptor is also expressed in glial cells in lamina I and II dorsal horn of the spinal cord [45]. Capsaicin stimulates excitatory and inhibitory transmission at dorsal horn level [46,47]. In particular, TRPV1 receptor stimulation induces the release of substance P, which in turn excites inhibitory neurons in laminae I, III and IV resulting in the enhancement of GABA- and glycinergic inhibitory post synaptic currents (IPSCs)[48-50].

## TRPV1 receptor and inflammatory pain

Phosphorylation and dephosphorylation reactions regulate TRPV1 receptor activity and proved to be crucial in promoting inflammatory pain. Activation of TRPV1 receptor during inflammation appears to be a dynamic process that is produced by the combination of endogenous ligands, phosphorylation, low pH and body temperature. Pro-inflammatory agents such as nerve growth factor (NGF), ATP, bradykinins, serotonin, histamine, proteases and chemokines lead to TRPV1 sensitization through phospholipase C activation. PLC-induced sensitization reduces the threshold for detecting painful stimuli during inflammatory pain [51-54].

TRPV1-/- mice have shown markedly decreased thermal hyperalgesia in inflammatory pain models, confirming that the sensitization of these channels is likely to be involved in this phenomenon [55,56]. TRPV1 receptor activity is also modulated by phosphatidylinositol-4,5-bisphosphate (PIP2) via the activation of phospholipases like PLC. Two opposing effects have been reported on TRPV1 receptor activity by PIP2. It can both activate and inhibit TRPV1 receptor depending on the experimental conditions, or more specifically on the level of stimulation of the channel. PIP2 plays a role in desensitization of TRPV1 receptor; a condition that causes TRPV1 activation-induced currents decay during prolonged activation. Calcium flowing through TRPV1 channel activates a Ca^2+^-sensitive PLC, which hydrolyzes PIP2 and leads to its depletion, which in turn results in diminished channel activity. TRPV1 channel currents require PIP2 or other phosphoinositides since they may be inhibited by scavenging endogenous phosphoinositides with polylysine and reactivated by the application of PIP2 or other phosphoinositides [57,58]. Nonetheless, it has also been discovered that after exposure of TRPV1 receptor to high capsaicin concentrations, the ensuing Ca^2+ ^influx activates PLC, which results in the depletion of PIP2 and PtdIns(4)P, which in turn reduces channel activity leading to desensitization [57]. It has been suggested that the balance between the inhibitory and facilitatory effects of PIP2 on TRPV1 receptor depends on the stimulation level of the channel, since during sensitization PLC-coupled agonists induce a moderate depletion of PIP2, removing its inhibitory effect, although not producing low enough lipid levels to inhibit channel activity. In contrast, high capsaicin concentrations induce a severe depletion of PIP2 that limits channel activity and leads to desensitization [57]. On this subject, TRPV1 receptor regulation by lipids seems to be anything but simple. Moreover, it has recently been suggested that ethanol enhances TRPV1 mediated responses via PIP2-TRPV1 receptor interaction [58]. Phosphoinositide 3-kinase also modulates TRPV1 activity by facilitating TRPV1 receptor trafficking to the plasma-membrane in response to NGF released during inflammation, leading to hyperalgesia (see forward section) [59]. Other membrane-derived lipids also regulate TRPV1 receptor activity. Prostaglandin E2 (PGE2) and prostacyclin I2 (PGI2) enhance capsaicin-induced current in DRG [60] and reduce the temperature threshold for TRPV1 receptor activation [61]. PGE2 increases cAMP levels and therefore activates PKA, which directly phosphorylates the channel [62] within Thr 144, Thr 370, and Ser 502 residues which have been implicated in sensitization of heat-evoked TRPV1 receptor responses, suggesting a role for PKA in the development of thermal hyperalgesia [63]. Oleylethanolamide (OEA), a natural analogue of the endogenous cannabinoid anandamide, anandamide itself and some lipoxygenase products such as 12- and 15-HPETEs (hydroperoxyeicosatetraenoic acids) and 5- and 15-HETEs (hydroxyeicosatetraenoic acids) all positively modulate TRPV1 receptor function [17,64].

Chronic pain-induced sensitization also intensifies the expression of TRPV1 receptor in sensory neurons through transcriptional and translational regulation, post-translational changes and altered trafficking, contributing to the development of pathological pain states in which increased sensitivity to noxious stimuli occurs [65]. NGF retrograde transport increases the translation of TRPV1 receptor in the cell body during inflammation resulting in an increased translation of TRPV1 receptor to the cell body [66]. NGF also activates phosphoinositide 3-kinase (PI3K)-SRC kinase signalling pathway which phosphorylates intracellular stores of TRPV1 leading to its assembly within the cellular membrane [67].

## TRPV1 receptors and neuropathic pain

The nerve growth factor (NGF), which plays a major role in the development and maintenance of neuropathic pain following peripheral nerve injury [68] has been proven to up-regulate TRPV1 receptor [69] and be involved in the phenotypic changes which lead to the development of neuropathic pain. TRPV1 receptor is up-regulated in undamaged neurons and down-regulated in damaged ones in several models of neuropathic pain [70] and, interestingly, the increased expression of TRPV1 receptor is related not only to the C-fibers but also to the myelinated A-fibers, which justifies the effectiveness of TRPV1 receptor agonist/antagonists in mechanical allodynia, apart from thermal hyperalgesia. In the tight ligation of the L5 spinal nerve, another model of neuropathic pain, the expression of TRPV1 receptors in the injured L5 dorsal root neurons decreased, whereas it increased in the uninjured L4 dorsal root neurons [70]. The up-regulation of the TRPV1 receptor at peripheral and central nervous system level in neuropathic pain conditions provides morphological evidence that the sensitivity of the vanilloid system in this painful condition is increased (conversely to the opioid system), therefore making the TRPV1 channel a suitable candidate for the future development of novel neuropathic pain relieving agents. TRPV1 receptor up-regulation also appears to occur at central sites leading to an enhancement of glutamatergic signalling in the spinal cord [71]. Similarly to peripheral sites, sensitization associated with an up-regulation of spinal TRPV1 receptor is thought to be at the base of the development of mechanical allodynia in the chronic constriction injury of the sciatic nerve [72].

The increased expression of TRPV1 receptor in pathological pain conditions [73] and its desensitization property associated with stimulation presents a valid strategy for pain relief and opens up the perspective of curing currently untreatable conditions such as neuropathic pain. Topical application of capsaicin proved effective in different neuropathic pain conditions, including post-herpetic neuralgia [74] and surgical neuropathic pain [75] but ineffective in chronic distal painful polyneuropathy [76]. However, topical capsaicin treatment such as creams, lotions or patches is associated with irritation, discomfort and pain due to the activation of sensory neurons expressing TRPV1 receptors in humans. This factor has driven chemical synthesis towards novel vanilloids with an improved desensitization/pungency ratio. The mechanisms at the basis of the anti-hyperalgesic action of topically applied capsaicin in neuropathic pain induced by partial sciatic nerve injury have been investigated. The novel expression of TRPV1 receptors on neonatal capsaicin-insensitive fibers confirmed by immunohistochemistry accounted for the anti-hyperalgesic action of topical capsaicin [77]. TRPV1-/- mice show normal responses to noxious mechanical stimuli but exhibited no vanilloid-evoked pain behaviour, reduced detection of painful heat and limited thermal hypersensitivity in the setting of inflammation. TRPV1 channels appear to be essential to the selective detection of pain sensations and to thermal hyperalgesia associated with inflammatory pain [55,56]. Vanilloid application on sensory neurons causes pain symptoms such as thermal hyperalgesia and mechanical allodynia similar to those associated with neuropathic pain [78,79]. Indeed TRPV1 receptor blockers should prove to be analgesics in neuropathic pain conditions. Early studies using capsazepine, the prototype TRPV1 antagonist, failed to produce any analgesic effect in models of acute and chronic pain in rats [80]. Subsequently, studies using capsazepine have shown that this drug inhibited noxious heat, protons and capsaicin-induced responses on cloned human [81] or guinea pig [82] TRPV1 receptors, whereas it failed to inhibit the responses to low pH on rat TRPV1 receptors, thus indicating possible species differences in the pharmacology of TRPV1 receptors. Interestingly, capsazepine reversed hind paw capsaicin-, CFA- and carrageenan-induced thermal hyperalgesia and mechanical allodynia in models of inflammatory and neuropathic pain in the guinea pig [83]. Capsazepine was also effective in reversing partial sciatic nerve ligation-induced mechanical hyperalgesia in such rodents while it exhibited little analgesic effect in mice or rats with neuropathic and inflammatory pain. This species-specificity in capsazepine pharmacological action has led to the development of new, potent and selective TRPV1 antagonists. Pharmaceutical companies have been expressing a great interest in the development of potent and selective TRPV1 antagonists. A potent, selective, and orally bioavailable antagonist of rat TRPV1, the N-(4-tertiarybutylphenyl)-4-(3-chloropyridin-2-yl)tetrahydropyrazine-1(2H)-carbox-amide, BCTC, has been developed by Valenzano et al. [84] and tested in models of chronic pain in rats. BCTC exhibited pain relief in mechanical and thermal hyperalgesia induced by intraplantar injection of capsaicin or CFA. BCTC also reduced already established mechanical hyperalgesia and tactile allodynia 2 weeks after partial sciatic nerve injury, and did so with a safe side effect profile [85]. Another potent and selective antagonist of both human and rat TRPV1 receptors, the 1-isoquinolin-5-yl-3-(4-trifluoromethyl-benzyl)urea, A-425619, proved dose dependently effective in several models of inflammatory and postoperative pain. A-425619 showed efficacy after either oral and intrathecal administration or local injection into the inflamed paw. Furthermore, A-425619 also showed partial efficacy in models of neuropathic pain without altering motor performance [86]. Similarly, (E)-3-(4-t-butylphenyl)-N-(2,3-dihydrobenzo[b][1,4]dioxin-6-yl)acrylamide, AMG 9810, a competitive antagonist of TRPV1 receptors, has been shown to reverse thermal and mechanical hyperalgesia associated with CFA-induced inflammatory pain without any significant effect on motor function [87]. Two further potent TRPV1 receptor antagonists, one with high and the other with low CNS penetration (the 1-[3-(trifluoromethyl)pyridin-2-yl]-N-[4-(trifluoromethylsulfo-nyl)phenyl]-1,2,3,6-tetrahydropyridine-4-carboxamide, A-784168, and the N-1H-indazol-4-yl-N'-[(1R)-5-piperidin-1-yl-2,3-dihydro-1H-inden-1-yl]urea, A-795614, respectively), delivered through different administration routes (oral, intrathecal or intracerebroventricular), clarified that peripheral, spinal and supraspinal TRPV1 receptors are all involved in analgesic actions in inflammatory pain conditions. Moreover, centrally penetrating TRPV1 receptor antagonists proved more effective compared to peripheral restricted agents with the same pharmacokinetic and pharmacological profile [88], underlining an involvement of central TRPV1 receptor blockade in the analgesic action. A piperazinylpyrimidine analogous, AMG517, was shown to reverse inflammation-induced pain behaviour in rats, target central TRPV1 receptor and have a long half-life that may be amenable to a one-a-week administration [88,89]. However, during a double blind, placebo controlled, randomized, parallel group, multicenter study for the management of pain following molar extraction, TRPV1 receptor antagonists caused prolonged hyperthermia (> 40°C) after taking a 2 mg dose, thus revealing the importance of TRPV1 receptor in central core temperature regulation [90]

## TRPV1 receptor antagonists and body temperature

Most TRPV1 receptor antagonists described to date cause modest dose-limited increases in body temperature in preclinical studies [91]. Studies using TRPV1 knockout mice have unequivocally demonstrated that TRPV1 receptor antagonist-elicited hyperthermia is TRPV1 mediated [92]. Moreover, the magnitude and duration of temperature elevation caused by TRPV1 antagonists proved to be dependent on the specific properties (pharmacokinetic profile, specific modality of TRPV1 blockade) of the individual TRPV1 receptor antagonists [93,94]. Indeed, the temperature elevation caused by the clinical candidate TRPV1 receptor antagonist ABT-102 was modest (0.6°C) and transient [94] compared to that observed with AMG-517 (1.6°C) [93]. However, the fact that numerous structurally distinct compounds blocking TRPV1 receptor cause hyperthermia indicates that TRPV1 receptor is tonically active in controlling body temperature in non-pathological states [91,93]. Several efforts have been made in order to eliminate hyperthermia-related side effects from TRPV1 receptor blockade. Such efforts have led to the development of profile C modulators such as the (R,E)-N-(2-hydroxy-2,3-dihydro-1H-inden-4-yl)-3-(2-(piperidin-1-yl)-4 (trifluoromethyl)phenyl)acrylamide, AMG8562. AMG8562 blocks capsaicin activation of TRPV1 receptor, does not affect heat activation of TRPV1 receptor, potentiates pH 5 activation of TRPV1 receptor in vitro, and does not cause hyperthermia in vivo in rats. AMG8562 has been found to significantly block capsaicin-induced flinching behaviour, produces statistically significant efficacy in complete Freund's adjuvant- and skin incision-induced thermal hyperalgesia, and acetic acid-induced writhing model, with no profound effects on motor activity [95]. The hurdle of TRPV1 antagonist-hyperthermia therefore seems to be at a turning point.

## Towards hybrid compounds targeting TRPV1 and cannabinoid receptors

Several studies are emerging which highlight the fact that the analgesic effects of compounds that interact with the endocannabinoid system are also mediated by TRPV1 channels. This is due to the fact that some endovanilloids/endocannabinoids such as anandamide may activate TRPV1 and cannabinoid receptors. It is not surprising then that the effectiveness of AM404, an inhibitor of endocannabinoid cellular uptake, in alleviating neuropathic pain has been attributed (at least in part) to TRPV1 receptor stimulation [96]. Cannabidiol is a major component of *Cannabis Sativa *and its analgesic effect was prevented by capsazepine in a model of neuropathic pain [97]. The inhibition of the metabolism of endocannabinoids by blocking the enzyme fatty acid amide hydrolase, FAAH, proved to be effective in neuropathic pain [98]. FAAH inhibition, by elevating the levels of anandamide and other N-acylethanolamine besides cannabinoid involves TRPV1 receptors. It is worth noting that in a study carried out by Maione et al. [99], N-arachidonoyl-serotonin (AA-5-HT), a dual FAAH inhibitor and TRPV1 receptor blocker, proved to be analgesic after repeated administration in rats in the sciatic nerve ligation model of neuropathic pain. When compared to much more potent FAAH inhibitors (URB597 and OL135), AA-5-HT showed similar or even greater effectiveness, hence confirming the role of TRPV1 receptor blockade in alleviating symptoms of neuropathic pain. Therefore by simultaneously targeting FAAH enzyme and TRPV1 receptors, two different targets controlling nociception in distinct ways, this hybrid molecule could represent an alternative approach to be used in the treatment of neuropathic pain. Such a strategy might even solve the problems of the hyperthermia caused by some "pure" TRPV1 antagonists, since AA-5-HT does not cause such side effect (possibly because indirect activation of CB1 receptors might be the cause of hypothermia).

## Nociceptive fiber deletion by TRPV1 activation

As an alternative to TRPV1 receptor antagonism, therapeutic nociceptive cell deletion, exploiting the enriched TRPV1 receptor expression in nerve terminals of dorsal root or trigeminal ganglia, has been proposed as a strategic approach to the management of severe pain. In fact persistent activation of TRPV1 receptors induces a strong and prolonged increase in intracellular Ca^2+^, leading to excitotoxicity which compromises and deletes TRPV1-expressing cells [100]. Over-stimulation of TRPV1 receptor would prove useful in deleting TRPV1 receptor-positive neurons, thereby eliminating any sensitivity to nociceptive stimuli in hyperalgesic conditions such as inflammatory or neuropathic pain, without affecting normal sensory transmission involving fibers that do not express TRPV1 receptors. RTX application to dorsal root or trigeminal ganglia selectively ablates vanilloid-sensitive nociceptive neurons, while leaving other adjacent neurons unaffected. Such treatment blocked experimental inflammatory hyperalgesia and neurogenic inflammation in rats and naturally occurring cancer and debilitating arthritic pain in dogs. Interestingly, sensations of touch, proprioreception and high threshold mechanonociception were unaffected [101]. Similarly, in rats perineural RTX application to the sciatic nerve inhibited inflammatory hyperalgesia in a dose- and time-dependent manner, despite leaving proprioreceptive and nociceptive sensations and motor control unaffected [102]. In rats already exhibiting neuropathic pain, RTX injection into the dorsal root ganglia of the L3, L4, L5 and L6 nerve roots increased the withdrawal threshold showing that the TRPV1-positive neurons mediate the most sensitive part of mechanical allodynia. When RTX was administrated into the ipsilateral dorsal root ganglia before nerve injury, such treatment prevented the development of tactile allodynia in 12 out of 14 rats. Immunohistochemical staining revealed that the TRPV1 receptor positive neurons were eliminated in rats that did not develop tactile allodynia, whereas they were still present in the allodynic rats. RTX injection in sensory ganglia could therefore represent an effective and broadly applicable strategy for pain management in neuropathies [103].

## Towards supraspinal TRPV1 receptor and the activation of an antinociceptive descending system

TRPV1 receptors have been identified in various regions of the brain known for their role in pain transmission or modulation [104-106] such as RVM, PAG, amygdala, solitary tract nucleus, somatosensory cortex, anterior cingulated cortex and insula [107,108]. TRPV1 receptor has been found on astrocytes, perivascular structures and neuron cell bodies and dendrites mainly on postsynaptic spines [104,105,109-111]. Capsaicin evokes glutamate release from slices of hypothalamus in a Ca^2+^-dependent way [110]. Such an effect was proven to be inhibited by capsazepine, suggesting that TRPV1 receptor may be expressed on glutamatergic neurons in the hypothalamus [110].

An earlier role of supraspinal TRPV1 receptor on pain transmission and modulation was evidenced by intracerebroventricular (ICV) capsaicin injections which decreased nociceptive threshold and reduced morphine- and stress-induced analgesia [111,112]. Conversely, ICV capsazepine or ruthenium red, another TRPV1 receptor antagonist, attenuated nocifensive behaviour induced by an intradermal injection of capsaicin or formalin in mice [113]. An effect of capsaicin involving supraspinal structures was also observed after systemic capsaicin administration. This treatment increased the firing activity of locus coeruleus (LC) neurons, an area which is activated by painful stimuli and whose stimulation produces antinociception even after sensory nerve fiber destruction [114,115]. Accordingly, TRPV1 receptor activation with capsaicin increased glutamatergic miniature excitatory postsynaptic currents in LC [116] and when injected into the ventral segmental area enhanced dopaminergic output to the nucleus accumbens [117].

Activation of the TRPV1 receptor also evokes glutamate release in the cortex [118]and capsaicin application to the somatosensory cortex reduced mechanically and electrically evoked potentials of anesthetized rats [119]and increased the firing rate of some neurons while depressing firing of other neurons in anterior cingulate cortex, an area involved in pain-related memory and descending modulation of nociception [120-122].

In one of our earlier studies, the intra-dorsolateral (DL) PAG microinjection of capsaicin increased the latency of the nociceptive reaction in the plantar test. This analgesic effect required glutamate release and subsequent activation of glutamate receptors such as group I metabotropic glutamate (mGlu) and N-methyl-D-aspartate (NMDA) receptors. Indeed riluzole, a voltage-dependent Na^+ ^channel blocker that enhances the uptake of glutamate, mGlu subtypes I and 5 (mGlu1 and mGlu5) and NMDA receptor antagonists blocked the analgesic effect induced by capsaicin [123]. We proposed that capsaicin-induced antinociception was due to the activation of the descending antinociceptive pathway via a TRPV1 receptor-dependent increase in glutamate release and the downstream activation of postsynaptic mGlu1/5 and NMDA receptor. The microinjection of capsaicin within the DL PAG induced brief hyperalgesia followed by analgesia in another study where a higher concentration of capsaicin was used [124]. TRPV1 receptor activation and the consequent desensitization may have been responsible for the observed effects, due to the fact that sustained activation of TRPV1 receptor leads to its desensitization. In another of our studies intra-VL PAG administration of URB597, an inhibitor of FAAH, by raising the endocannabinoid level resulted in a dose-dependent dual effect on pain responses depending on TRPV1 or CB1 receptor stimulation [125]. Indeed, the different recruitment of CB1 or TRPV1 receptors by the increased endocannabinoid/vanilloid level may lead to hyperalgesia or analgesia. Hyperalgesia was proposed to be due to CB1 receptor stimulation which leads to inhibition of the antinociceptive PAG-RVM descending pathway. Higher doses of URB597 caused rapid analgesia blocked by capsazepine, being due to TRPV1 receptor stimulation. Therefore, by acting on either CB1 or TRPV1 receptor within the PAG, endocannabinoids lead to pronociceptive and antinociceptive effects through an inhibitory or facilitatory action on the pain descending pathway. Consistently, the same study showed that some neurons within the PAG coexpressed TRPV1 and CB1 receptors.

The intra- VL PAG microinjection of the FAAH and TRPV1 receptor blocker, AA-5-HT, induced analgesia in several pain models, such as tail flick, plantar and formalin tests. AA-5-HT also depressed the RVM OFF cell as well as ON cell activity. RVM ON and OFF cells represent an electrophysiological method for investigating the analgesic potential of drugs acting centrally. Indeed, morphine causes an increase in the activity of OFF cells (which are defined as antinociceptive) and an inhibition of the ON cells (pronociceptive). Being analgesic, AA-5-HT should have induced an increase in the OFF cell activity. The effect of AA-5-HT was mimicked by co-injecting the selective FAAH inhibitor URB597 (which blocks FAAH activity) in combination with I-RTX (which blocks TRPV1 receptors). Accordingly, analgesia was induced together with inhibition of ongoing ON and OFF cell activities. The recruitment of an ''alternative'' pathway, identified as PAG-locus coeruleus (LC)-spinal cord was attributed to the AA-5-HT-induced effect. Indeed, intra-VL PAG AA-5-HT increased LC neuron firing activities while intrathecal phentolamine or ketanserin prevented the analgesic effect of AA-5-HT. Moreover, intra-PAG AA-5-HT prevented the changes in the ON and OFF cell firing activity induced by intra-paw formalin, and it reverted the formalin-induced increase in LC adrenergic cell activity. All AA-5-HT effects were antagonized by cannabinoid CB1 and TRPV1 receptor antagonists, thus suggesting that co-localization of these receptors in the PAG could be an appropriate neural substrate for AA-5-HT-induced antinociception [126].

The direct stimulation of TRPV1 receptors by microinjection of capsaicin into the VL PAG increased the latency of the nociceptive reaction [127] as it did in the DL-PAG [123]. The antinociceptive effect of capsaicin when microinjected into the VL PAG was accompanied by an increase in glutamate release in the RVM. Blockade of TRPV1 receptors by I-RTX prevented capsaicin-induced antinociception and the increase in RVM glutamate release. At a higher dose, I-RTX facilitated nociceptive response and lowered the release of glutamate, suggesting that within the VL PAG, TRPV1 receptors tonically stimulate glutamatergic output to the RVM, and concomitantly inhibit nociception [127]. The VL PAG shows a high density of TRPV1 receptor positive profiles with strong immunoreactivity mostly found in cell bodies. The high density of vesicular glutamate transporter 1 (VGLUT1) on nerve terminals surrounding TRPV1 receptor positive cells may indicate glutamatergic input on TRPV1-expressing cells. In the VL PAG, many fibers were also vesicular GABA transporter (VGAT) positive, and VGAT-immunoreactivity was observed around TRPV1 receptor positive cells, demonstrating that TRPV1-expressing neurons could also receive GABAergic inputs [127]. In accordance with behavioral studies, McGaraughty et al. [124] found that microinjection of capsaicin into the DL PAG produced an initial activation, followed by a decrease, in the tail flick related ON-cell burst of activity and an increase in OFF-cell spontaneous firing.

Microinjection of capsaicin into the VL PAG caused a decrease in the firing activity of the ON cells and a very rapid increase in the firing activity of the OFF cells. Conversely, intra-VL PAG I-RTX increased the firing activity of ON cells while decreasing the firing activity of the OFF cells. Intra-VL PAG capsaicin also decreased tail flick-induced ON cell peak firing and delayed the onset of OFF cell pause. Thus, stimulation of TRPV1 receptor within the DL and VL subregion of the PAG seems to lead to activation of the antinociceptive OFF cells and inhibition of the pronociceptive ON cells, consistent with the behavioural analgesia. These effects were prevented by pre-treatment with I-RTX at a dose which did not significantly change the ongoing RVM ON and OFF cell activities per se. At a higher dose, I-RTX increased the duration of the OFF cell pause and shortened its onset [127].

As a whole, this evidence shows that pharmacological manipulation of TPRV1 receptor within the PAG may be a suitable strategy to activate the antinociceptive PAG-RVM-dorsal horn circuitry for producing analgesia [see 128 for review].

## Does TRPV1 receptor have a role in microglia/astrocytes-mediated synaptic plasticity?

Until recently, pain had been thought to arise primarily from the dysfunction of neurons. Recent evidence, however, suggests that neuro-immune changes might also contribute to pain following injury to the nervous system. Glial cells involved in mediating inflammatory processes are resident within the spinal cord and include both astroglia and microglia, the latter of which has been directly implicated in the initiation of peripheral injury-induced pain [129]. Moreover, microglia have been shown to express cannabinoid receptors [130] and to produce and inactivate endocannabinoids [131,132]. In addition, it has been proposed that TRPV1 receptor activation mediates microglial cell death in vivo and in vitro via Ca^2+^-mediated mitochondrial damage and cytochrome C release [133] and that TRPV1 receptor in astrocytoma cells can contribute to apoptosis via Ca^2+ ^influx and the activation of p38 [134]. Thus, besides the crucial role of TRPV1 channels in sensory neurons, where they regulate acute thermal nociception and inflammatory hyperalgesia, the investigation of the role of these receptors on glia and microglia could provide a new tool in further investigation into chronic diseases such as neuropathic pain. In fact, a contribution of TRPV1 receptors in glia and microglia activation has been demonstrated at the spinal level in mice with acute, inflammatory and neuropathic pain. In particular, it has been suggested that TRPV1 receptor-knockout mice show a higher density of astrocytes and microglia positive profiles under normal conditions than their wild-type littermates. Inflammatory or neuropathic pain induced a increase in glia and microglia in wild- type than in knockout mice, although the latter showed increased glial immunoreactivity as compared to untreated/uninjured animals. Thus, TRPV1 receptor could be involved in activating spinal glia in mice in different pain models including neuropathic pain and may have varying underlying mechanisms at different stages during the progression of the pain state [135]. However, the role of TRPV1 channels in glial and microglial phenotypical changes and at supraspinal level needs and deserves further investigation.

## Conclusion

The few findings about the role of supraspinal TRPV1 receptors in controlling pain appear encouraging. The possible exploitation of TRPV1 receptor for activating the antinociceptive descending pathway at PAG level may be a strategy of interest. Indeed a direct agonist such as capsaicin, an indirect one such as URB597 or an hybrid anadamide hydrolysis and TRPV1 receptor blocker such as AA-5-HT, lead to analgesia. TRPV1 receptor stimulation is associated with increased glutamate release and activation of group I mGlu and NMDA receptors and with modulation of downstream RVM ON and OFF neurons. Immunohistochemical data strongly support the hypothesis that the activation/inhibition of TRPV1 receptor expressed on glutamatergic neurons following intra-PAG injection of capsaicin/I-RTX leads to an increase/decrease in glutamate release and behavioural analgesia/hyperalgesia. Exogenous compounds capable of activating TRPV1 channels or strategy increasing *e*ndovanilloids within the PAG-RVM counteract nociception. Thus, TRPV1 receptor (and hopefully other TRP channels such as TRPA1 and TRPM8 which often co-localize with TRPV1 on peripheral nerve terminals) may offer a suitable target for enhancing the activity of the endogenous antinociceptive descending pathway and producing analgesia. Moreover, hybrid molecules with a dual mechanism, such as AA-5-HT, which blocks FAAH activity and TRPV1 receptors, do not induce hyperthermia as "classic" TRPV1 antagonists do. As well as the PAG-RVM antinociceptive axis, other regions of the brain known to influence descending pain modulation, such as the amygdala, thalamus and LC show TRPV1 receptor localization, although the exact contribution of such receptors, if any, has yet to be determined. Further studies to clarify the role of the supraspinal TRPV1 receptor and in particular, within the antinociceptive descending pathway, may open the way to a novel strategy of pain relief in central nervous system disorders, such as neuropathic pain, which does not yet have an appropriate therapy.

## Competing interests

The authors declare that they have no competing interests.

## Authors' contributions

EP has written the manuscript. EP and SM have conceived and conceptualized the manuscript. LL has written the paragraph on microglia. VdN, LB and FR contributed to the drafting of the paper. All authors have read and approved the final manuscript.
